# Inhibitor of DNA-Binding Protein 4 Suppresses Cancer Metastasis through the Regulation of Epithelial Mesenchymal Transition in Lung Adenocarcinoma

**DOI:** 10.3390/cancers11122021

**Published:** 2019-12-14

**Authors:** Chi-Chung Wang, Yuan-Ling Hsu, Chi-Jen Chang, Chia-Jen Wang, Tzu-Hung Hsiao, Szu-Hua Pan

**Affiliations:** 1 Graduate Institute of Biomedical and Pharmaceutical Science, College of Medicine, Fu Jen Catholic University, New Taipei City 242, Taiwan; 075006@mail.fju.edu.tw; 2 Graduate Institute of Medical Genomics and Proteomics, College of Medicine, National Taiwan University, Taipei 100, Taiwanchiajen.wang@gmail.com (C.-J.W.); 3 School of Medicine, College of Medicine, Fu Jen Catholic University, New Taipei City 242, Taiwan; jorge@seed.net.tw; 4 Division of Pediatric Surgery, Department of Surgery, Shin Kong Wu Ho-Su Memorial Hospital, Taipei 111, Taiwan; 5 Cell Therapy Center, Chang Gung Memorial Hospital, Taoyuan 333, Taiwan; 6 Department of Medical Research, Taichung Veterans General Hospital, Taichung 407, Taiwan; d93921032@gmail.com; 7 Doctoral Degree Program of Translational Medicine, National Taiwan University, Taipei 100, Taiwan; 8 Genome and Systems Biology Degree Program, National Taiwan University and Academia Sinica, Taipei 100, Taiwan

**Keywords:** Id4, lung cancer, EMT, MET, metastasis

## Abstract

Metastasis is a predominant cause of cancer death and the major challenge in treating lung adenocarcinoma (LADC). Therefore, exploring new metastasis-related genes and their action mechanisms may provide new insights for developing a new combative approach to treat lung cancer. Previously, our research team discovered that the expression of the inhibitor of DNA binding 4 (Id4) was inversely related to cell invasiveness in LADC cells by cDNA microarray screening. However, the functional role of Id4 and its mechanism of action in lung cancer metastasis remain unclear. In this study, we report that the expression of Id4 could attenuate cell migration and invasion in vitro and cancer metastasis in vivo. Detailed analyses indicated that Id4 could promote E-cadherin expression through the binding of Slug, cause the occurrence of mesenchymal-epithelial transition (MET), and inhibit cancer metastasis. Moreover, the examination of the gene expression database (GSE31210) also revealed that high-level expression of Id4/E-cadherin and low-level expression of Slug were associated with a better clinical outcome in LADC patients. In summary, Id4 may act as a metastatic suppressor, which could not only be used as an independent predictor but also serve as a potential therapeutic for LADC treatment.

## 1. Introduction

Lung cancer is the leading cause of cancer-related death worldwide [1,2]. Delayed diagnosis and early metastasis are the major unsolved obstacles for most physicians in treating this disease. In general, lung cancer can be divided into two major groups called non-small cell lung cancer (NSCLC) and small-cell lung cancer (SCLC). The former accounts for 85% of lung cancer patients and close to 50% of this proportion are lung adenocarcinoma (LADC) patients. Despite advances in LADC treatment that have been made over the past decades, the molecular mechanism of this type of cancer has remained unclear [3]. Therefore, identifying novel genes and their mechanisms of action involved in LADC progression and metastasis may provide new insights into the pathogenesis and management of lung cancer treatment.

As we know, cancer metastasis is a complex process that includes many steps, such as the cells migrating away from the primary tumor, invading the circulation, and subsequently colonizing the distant organs [4,5,6,7,8]. Among them, epithelial–mesenchymal transition (EMT) plays a crucial role in the regulation of cancer metastasis. Usually, EMT is a developmental process that is silent in normal and healthy tissues [8,9]. However, reactivation of EMT may occur in several pathological conditions, such as wound healing, chronic inflammation, and cancer [9,10,11,12]. Conceptually, EMT allows cancer cells to dedifferentiate, and acquire migratory and invasive abilities, which then promote the malignancy of cancer. In the past, many gene expression changes-E-cadherin (E-cad), N-cadherin (N-cad), claudin, occludin, vimentin, etc.-have been observed in EMT processes [13,14,15,16]. Several signaling pathways related to cell adhesion, migration, invasion, and differentiation have also been found to be involved in the regulation of EMT execution [17]. Moreover, the cadherin switch also plays an important role [18]. Until now, many EMT core factors, including Snail, Slug, Twist, ZEB1, ZEB2, TCF3/E47, etc., have been identified [15,17]. The inhibitor of DNA binding 4 (Id4) protein is a major component that participates in the regulation of TCF3/E47.

Id4 is a member of the inhibitor of DNA binding proteins (Id proteins), which are key regulators in developmental and cellular processes [19,20,21,22,23]. Owing to the lack of a basic DNA-binding domain, Id proteins usually form heterodimers with a basic helix-loop-helix (bHLH) transcription factors through the HLH-dimerization domain and inhibit the DNA binding of bHLH proteins [19,20,21,22,24]. In mammals, four Id proteins, Id1–4, have been found. All of them share a highly homologous HLH region, whereas the rest of the sequences diverge among the members [21,25]. Although lots of the Id protein partners have been identified [25,26,27,28], the Id1–E-protein, Id–Ets, and Id2–Rb interactions are the most compelling [21,29,30,31,32]. Based on experiments with Id3, it has been proposed that the E proteins may chaperone the Id proteins into the nucleus and increase the half-lives of otherwise unstable proteins [33].

Recently, numerous studies indicated that Id proteins might be dysregulated in a variety of human cancers [23,34,35]. In astrocytic tumors, Id1–Id3 are highly expressed in higher graded tumor specimens [36]. In prostate cancer, the expression of Id1 was shown to be positively correlated with a poorly differentiated histology [37]. Additionally, the overexpression of Id2 might lead to polyclonal lymphomas in thymocytes [38]. Although Id4 has also been shown to participate in tumor progression, its functional role remains ambiguous [39,40]. In prostate cancer, Id4 acts as a tumor suppressor that increases cell apoptosis and inhibits cell proliferation through arresting S-phase progression [41,42]. However, in breast cancer, Id4 has been determined to be an oncogene, which promotes tumor neo-angiogenesis by the regulation of IL-8 and Gro-α [43]. Recently, scientists also found that Id4 is involved in the suppression of matrix metalloproteinase 2 (MMP2)-mediated cell invasion in glioblastoma [44] and also in the modulation of miR-342-regulated breast cancer type 1 susceptibility protein (BRCA1) expression [45]. As Id proteins play different roles in various cancers, the identification of how Id4 is involved in lung cancer metastasis has become an urgent need.

Previously, our research team performed cDNA microarray screening and identified that Id4 is one of the differentially expressed invasion-associated genes in a panel of lung cancer cells (CL1-0, CL1-1, and CL1-5) [46,47]. Herein, we explore the functional role of Id4 in cancer metastasis. Our data not only demonstrate that Id4 may suppress the malignant behavior of lung cancer through EMT regulation but also provide evidence to translate it for the clinical application of lung adenocarcinoma treatment in the near future.

## 2. Results

### 2.1. Id4 Expression Inversely Correlates with Lung Cancer Cell Invasiveness

**Figure 1:**
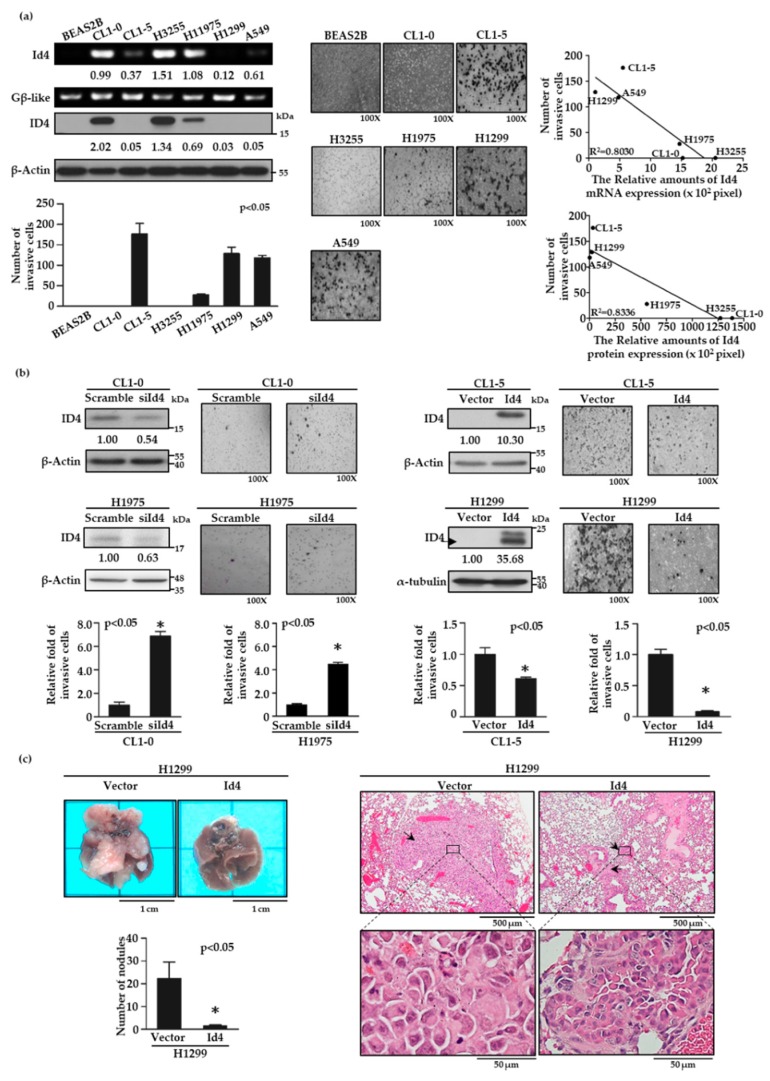
Inhibitor of DNA binding 4 (Id4) expression inversely correlates with lung cancer metastasis in vitro and in vivo. (**a**) Id4 mRNA and protein expression levels in different lung cancer cell lines were detected by RT-PCR (left, Id4) and immunoblotting (left, ID4). The numbers under the images of bands indicate the quantification of mRNA and protein expressions, both of which were calculated by ImageJ software and normalized to the internal control, Gβ-like or β-actin, of each cell line. The invasive ability of each cell line was evaluated by a modified Boyden chamber invasion assay in vitro. The images of the invasion assay (original magnification, ×100) were presented (middle) and the numbers of invasive cells were calculated (bottom left; *p* < 0.05 by one-way ANOVA). The correlation of Id4 expressions and cell invasiveness in different lung cancer cells was calculated by linear regression (top right: the correlation of Id4 mRNA expression and cell invasiveness; bottom right: the correlation of Id4 protein expression and cell invasiveness; *p* < 0.05). (**b**) Expressions of Id4 interfere with cell invasiveness. Id4 expressions and images of invasive cells (original magnification, ×100) are shown for CL1-0 or H1975/Id4-silencing (up, left) and CL1-5 or H1299/Id4-overexpressing (up, right) stable cell lines. The protein expression levels and the invasive abilities of Id4 stable cells were quantified. The relative fold changes compared with the control cells (* *p* < 0.05) are displayed. (**c**) The effects of Id4 expression in cancer metastasis in vivo were examined by a tail vein metastasis assay with H1299/Id4-overexpressing stable cells. The numbers of metastatic tumor nodules were calculated from five mice per group (* *p* < 0.05). Histology of the metastatic pulmonary nodules was confirmed as lung adenocarcinoma (LADC) by H&E staining; the arrows indicated the distribution of tumors, and the area of black rectangles was zoomed and presented at the bottom.

To characterize the role of Id4 in lung cancer invasiveness, we first examined the expression levels of Id4 in low invasive CL1-0 and high invasive CL1-5 cells by reverse transcription polymerase chain reaction (RT-PCR) and immunoblotting. As with the finding of the cDNA microarray (Figure S1a), both mRNA and protein expression levels of Id4 were inversely correlated with cell invasiveness (Figure S1b, *p* < 0.05 and Figure S5). Then, the negative correlation between Id4 expression and cell invasiveness was re-evaluated by four additional lung cancer cell lines, including H3255, H1975, H1299, and A549 cells, and a normal bronchus epithelial cell, BEAS-2B. As expected, both the mRNA and protein expression levels of Id4 were negatively correlated with cell invasiveness in different lung cancer cells (Figure 1a; R^2^ = 0.8336 for Id4 protein expression versus cell invasiveness, and 0.803 for Id4 mRNA expression versus cell invasiveness; and Figure S6a,b).

### 2.2. Expression of Id4 could Interfere with the Malignant Behavior of Lung Cancer Cells In Vitro and In Vivo

To further investigate the role of Id4 in cancer metastasis, we established Id4 silencing and overexpressing stable cells and examined their cell invasiveness by modified Boyden chamber invasion assays. The results showed that silencing the expression of Id4 in CL1-0 and H1975 cells could significantly increase the cell invasive ability compared with the scrambled control cells (Figure 1b, left, *p* < 0.05 and Figure S6c, left). Conversely, the overexpression of Id4 inhibited cell invasiveness in both CL1-5 and H1299 lung cancer cells compared with the vector control group (Figure 1b, right, *p* < 0.05 and Figure S6c, right). Next, we assessed whether Id4 could inhibit cancer metastasis in vivo. Id4-overexpressing H1299 lung cancer cells were injected into the tail veins of mice, and the formation of metastatic pulmonary nodules was examined for 10 weeks. As the observation in vitro, H1299/Id4-overexpressing cells resulted in fewer pulmonary nodules than those injected with H1299/vector control cells (Figure 1c, left; mean number of nodules, 1.50 ± 0.37 for H1299/Id4 and 22.2 ± 6.48 for H1299/vector; *p* < 0.05). The morphology of the metastatic lung nodules was recognized and displayed as LADC through hematoxylin and eosin (H&E) staining (Figure 1c, right). Moreover, a similar experiment was executed by CL1-5/Id4-overexpressing lung cancer cells and re-confirmed the inhibitory role of Id4 in lung cancer metastasis in vivo (Figure S2).

To rule out the possibility that the inhibitory effects of Id4 on cancer metastasis occurred through interfering with cell growth, we examined the effects of Id4 expression on cell proliferation and apoptosis in vitro and in vivo (Figure S3). The in vitro studies presented that nonsignificant differences existed for the growth rates of CL1-0/Id4-silenced and CL1-5/Id4-overexpressing stable cells compared with the scrambled and vector control cells respectively (Figure S3a). Although the apoptotic cell population of Id4-overexpressing stable cells was slightly increased compared to that of the vector control cells (Figure S3b; 2% of CL1-5/Id4-overexpression stable cells versus 0.9% of CL1-5/vector control cells), the changes did not reach significance. Next, we performed in situ examination by lung tissue specimens from mice metastatic pulmonary nodules to determine the cell proliferation and apoptosis in vivo. The data revealed that the expressions of Ki67, a well-known marker for cell proliferation, in cell nucleus did not display dramatic changes between the H1299/Id4-overexpressing group compared with that of the vector control group (Figure S3c). Simultaneously, the lung tumor tissues from both H1299/Id4-overexpressing and H1299/vector control cells exhibited slight apoptosis in the nucleus that was identical to the findings in vitro (Figure S3d). Collectively, we concluded that the expression of Id4 is inversely related to the regulation of cancer metastasis in vitro and in vivo.

### 2.3. Id4 Affects the Malignancy of Cancer Cell through the Regulation of Epithelial–Mesenchymal Transition

**Figure 2:**
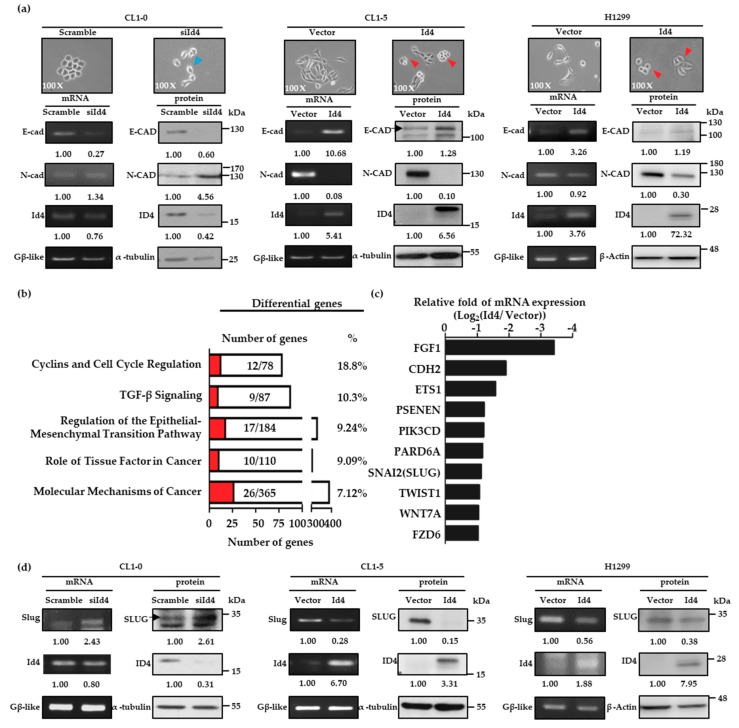
Id4 is involved in the regulation of epithelial–mesenchymal transition (EMT). (**a**) Cell morphology of CL1-0/Id4-silencing (left), CL1-5/Id4-overexpressing (middle), and H1299/Id4-overexpressing (right) cells was pictured, and the mRNA and protein expressions of two well-known EMT markers, E-cad and N-cad, were detected by RT-PCR and immunoblotting (bottom). The mRNA and protein expression levels in Id4-overexpressing or silencing cells were quantified, and the relative fold changes compared with the scramble or vector control cells are displayed. The arrow heads in the images indicate that the cells presented mesenchymal (blue arrows) or epithelial-like phenotypes (red arrows). (**b**) Biological functional analysis of differentially expressed genes by Ingenuity Pathway Analysis (red: the number of genes which was displayed changed at least twofold between CL1-5/Id4-overexpressing and CL1-5/vector control cell lines). (**c**) Top 10 genes significantly down-regulated in CL1-5/ Id4-overexpressing stable cells compared with those in CL1-5/vector control cells. (**d**) The mRNA and protein expression levels of Slug in CL1-0/Id4-silencing (left), CL1-5/Id4-overexpressing (middle), and H1299/Id4-overexpressing stable cell lines (right) were detected by RT-PCR and immunoblotting. The mRNA and protein expression levels in Id4-overexpressing or silencing cells were quantified and displayed the relative fold changes compared with the scramble or vector control cells.

Since the expression levels of Id4 could interfere with cancer metastasis, the next aim was to understand how Id4 is involved in this complex process. The morphology of the Id4 stable expressing lung cancer cells was first examined in detail, and we found that the CL1-0/Id4-silencing stable cells exhibited a spindly, elongated, and dispersed morphology, which are more like the mesenchymal-type characteristics, compared with the scrambled control cells. On the other hand, the CL1-5 and H1299/Id4-overexpressing cells exhibited an epithelial-like appearance and tended to form multicellular aggregates compared with the vector control cells (Figure 2a, upper panel). The morphological changes led us to hypothesize that the effect of Id4 on cancer metastasis is related to the event of epithelial-mesenchymal transition (EMT).

As such, we examined the expressions of E-cadherin (E-cad) and N-cadherin (N-cad), two well-known EMT markers, in CL1-0/Id4-silencing, CL1-5 and H1299/Id4-overexpressing stable cells by RT-PCR and immunoblotting. The results indicated that both mRNA and protein expressions of E-cad and N-cad were dramatically changed with the manipulation of Id4 expressions in CL1-5 and H1299/Id4-overexpressing stable cells; and the obvious changes of E-cad and N-cad protein expression could be also seen in CL1-0/Id4-silencing stable cells (Figure 2a, bottom panel; Figures S7 and S8). This suggests that Id4 may interfere with the malignant behavior of lung cancer cells through EMT regulation. Next, we used CL1-5/Id4-overexpressing stable cells to perform Affymetrix microarray analysis and explore the potential signaling that may be involved in Id4-induced mesenchymal-epithelial transition (MET). Differentially expressed genes were identified between CL1-5/Id4-overexpressing and CL1-5/vector control cells and clustered into up and down-regulated groups. The Ingenuity Pathway Analysis (IPA) showed that most of the differentially expressed genes were involved in cancer progression, the EMT processes, cell cycle regulation, etc. (Figure 2b).

By further examining the mRNA expression levels of the 17 targeted genes involved in EMT regulation, we found that 10 targeted genes’ mRNA expressions were significantly down-regulated (at least twofold changes) in CL1-5/Id4-overexpressing stable cells compared with CL1-5/vector control cells (Figure 2c). Among these genes, we were excited to find that SNAI2 (also called Slug), a well-known transcription repressor and an invasion enhancer, existed in our candidate pool. Therefore, we explored whether the expression of Slug could be interfered with by the manipulation of Id4 expression in lung adenocarcinoma cells. As expected, the mRNA and protein expression levels of Slug could be regulated by Id4 expressions in CL1-0, CL1-5, and H1299 lung cancer cells (Figure 2d, Figures S7 and S8). In addition, the positive and negative correlations between Id4, E-cad, and Slug protein expressions were re-confirmed by immunohistochemically staining using mice tissue specimens from the metastatic pulmonary nodules that were derived by H1299/Id4-overexpressing cells. As predicted, the tissue specimen derived from H1299/vector control cells only presented Slug expression in nucleus without signals of Id4 in the nucleus and E-cadherin (E-cad) in the cell membrane (Figure S4, up panel). On the other hand, the expression of E-cad in the cell membrane and Id4 in nucleus were both observed in the tissues of H1299/Id4-overexpressing cells without any signals of Slug in the nucleus (Figure S4, bottom).

### 2.4. Id4 Promotes E-cad Expression through the Binding of Slug

**Figure 3:**
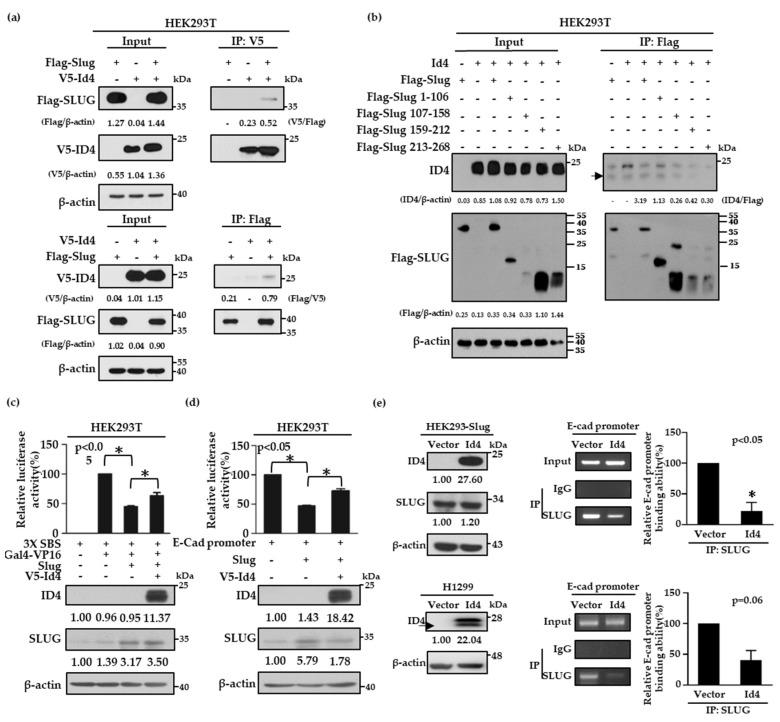
Id4 binds to Slug and inhibits the transcriptional repression activity of Slug in cells. (a,b) HEK293T cell lines were co-transfected with V5-tagged Id4 and Flag-tagged Slug (**a**) or different Slug mutants, which involved amino acid residues 1–106, 107–158, 159–212, and 213–268 (**b**), and the protein–protein interaction between Id4 and Slug or Slug mutants was recognized by immunoprecipitation with anti-V5 or anti-Flag antibodies (as indicated). In total, 30 μg total cell lysates were taken as the input. Black arrow: the molecular weight of ID4. (c,d) HEK293T cells were co-transfected with the indicated plasmids and a reporter vector driven by SBS-Gal4 (**c**) or by the E-cadherin promoter (**d**). Luciferase activities and immunoblotting were evaluated 24 h after transfection. The activity induced by Gal4-VP16 alone (**c**) or basal activity (**d**) was normalized to 100%. All data were reported as mean values ± SEMs, and *p*-values were calculated via Student’s *t*-test. The asterisk represents a *p* value of < 0.05 compared to the group stimulated with Gal4-VP16 alone or the basal activity level. The protein expression levels in cells were quantified and displayed the relative fold changes compared with the control cells, which were only transfected with promoter-construction. (**e**) Right: The IgG and Slug antibodies were used to pull down protein-DNA complexes in HEK293-Slug/Id4 and H1299/Id4 cell lines, and the E-cadherin promoter level in each sample was determined by PCR using a gene-specific primer set (bottom, *p* < 0.05). Input: an aliquot of each sample was prepared and used as a template for PCR to examine the level of the E-cadherin promoter before immunoprecipitation (IP). Left: Immunoblot analysis of the indicated proteins was performed on the products of the chromatin immunoprecipitation assay (ChIP). The protein expression levels in Id4-overexpressing cells were quantified and displayed the relative fold changes compared with the vector control cells.

As Slug is a well-known transcription repressor of E-cad and the expression of Id4 is correlated with both E-cad and Slug protein expressions, we were curious about whether Id4 is involved in the regulation of the Slug/E-cad axis. To address this question, V5-tagged Id4 and Flag-tagged Slug plasmids were first co-transfected into HEK293T cells to examine whether Id4 can interact with Slug proteins by immunoprecipitation. The data showed that V5-tagged Id4 and Flag-tagged Slug proteins could interact with each other, no matter whether a V5 antibody (Figure 3a and Figure S9a, up panel) or Flag antibody (Figure 3a and Figure S9a, bottom panel) was used to perform the immunoprecipitation assays. Next, we generated different fragments of Slug, including residues 1–106, 107–158, 159–212, and 213–268, to determine the reciprocal domain of Slug that was responsible for interacting with Id4. The results showed that the protein–protein interaction significantly disappeared when V5-tagged Id4 cooperated with the Slug fragment 159–212 and 213–268. This suggests that fragments 1–106 and 107–158 of Slug were needed for the interaction with Id4 (Figure 3b and Figure S9b). As the responsibly binding region of Slug contains parts of the DNA binding domain, this led us to wonder whether the interaction between Id4 and Slug could interfere with the repression activity of Slug in E-cad transcription. To answer this, we analyzed the repression activities of Slug with or without V5-tagged Id4 expression by the Snail-binding site (SBS)-Gal4 promoter and E-cadherin promoter, respectively. Here, HEK293T cells were co-transfected with the SBS-Gal4-luciferase reporter and Gal4-VP16 activator expression plasmids, together with or without the Slug and V5-tagged Id4 plasmids. As previously reported, Slug repressed the expression of the reporter gene [48]. The results indicated that the promoter activity of SBS could be significantly repressed when Slug protein was expressed in cells, and the repression activity of Slug would be reversed to nearly 20% when Slug and V5-tagged Id4 were co-expressed in cells (Figure 3c, *p* < 0.05; Figure S10a); the transfection efficacy of Slug and V5-Id4 was evaluated by immunoblotting. Simultaneously, this finding was re-confirmed using the E-cad promoter region, a well-known downstream target of Slug [49,50], and it was shown that the expression of Id4 could reverse the repression activity of Slug and promote E-cad promoter expression in cells (Figure 3d, *p* < 0.05; Figure S10b). Moreover, we also performed chromatin immunoprecipitation assays to confirm whether the reverse effect of Id4 on Slug repression activity occurs through inhibiting the binding of Slug on the E-cad promoter region in both HEK293-Slug/Id4 and H1299/Id4 stable cell lines. As predicted, the expression of Id4 could attenuate approximately 60–80% of the binding ability of the Slug protein to the E-cad promoter region no matter in HEK293-Slug or H1299/Id4 overexpressing stable cells (Figure 3e, *p* < 0.05; Figure S11). Collectively, all the findings suggest that Id4-induced E-cad expression is achieved through inhibiting the binding ability of the transcription repressor, Slug, on the E-cad promoter region.

### 2.5. Low-Level Id4 Expressions Are Associated with A Poor Clinical Outcome for LADC Patients

Although our results consistently suggested that cancer invasion could be counter-regulated by Id4 and Slug, both in vitro and in vivo, such studies did not fully reflect clinical malignancy. Accordingly, we extended our analysis by analyzing the gene expression data obtained from 168 pathological stage I lung adenocarcinomas in the public database GSE31210 [51]. The clinical characteristics of these NSCLC patients are summarized in Table S1.

**Figure 4:**
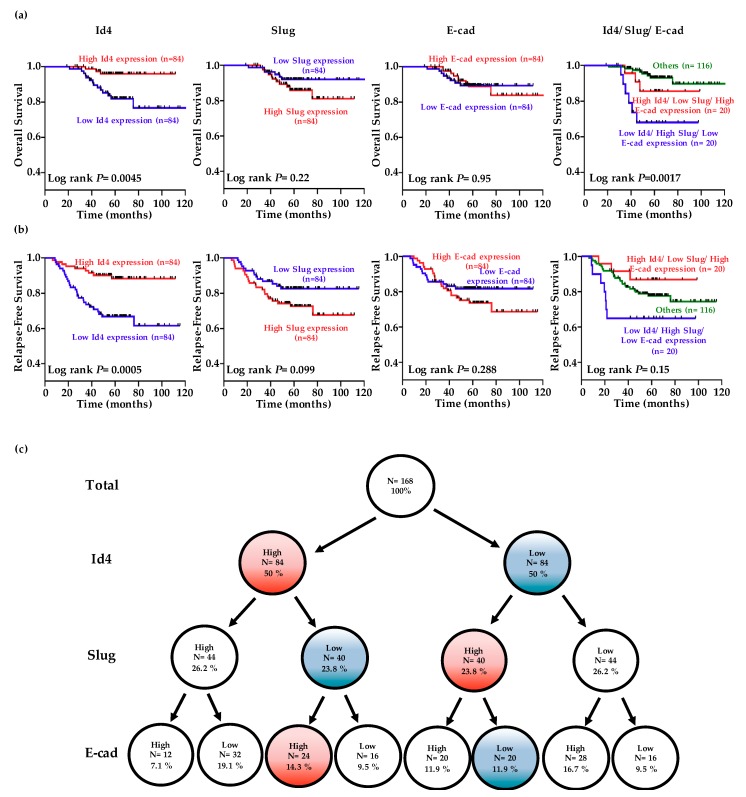
Low-level Id4 expression is associated with a poor clinical outcome in lung adenocarcinoma (LADC) patients. The data of Id4, Slug, and E-cadherin expression was obtained from 168 pathological stage I LADC patients in the public database GSE31210 [51]. (**a**,**b**) The patients were divided into the high- (red line) and low-expression (blue line) of each gene using the median value as the cutoff, and Kaplan-Meier estimates of overall survival and relapse-free survival in these patients. The combined effects of Id4, Slug, and E-cadherin on the overall survival and relapse-free survival of non-small cell lung cancer (NSCLC) patients were analyzed. *p*-values were calculated using the log rank test. (**c**) A tree diagram showing the proportions of patients with high and low Id4/Slug/E-cadherin expression levels in patients.

The levels of Id4, Slug, and E-cad were dichotomized into high or low expression categories using the median value as the cutoff. Kaplan-Meier analysis indicated that patients exhibiting lower Id4 expression levels experienced poorer overall survival and relapse-free survival than those displaying higher Id4 levels (Figure 4a,b; *p* = 0.0045 for overall survival and 0.0005 for relapse-free survival). Although patients exhibiting higher Slug expression levels seem experienced poorer overall survival and relapse-free survival than those displaying lower Slug levels, the *p*-values still not reach significance (*p* = 0.22 for overall survival and 0.099 for relapse-free survival). Analysis of the combined effect of Id4, Slug, and E-cad on patient prognosis revealed that patients displaying higher Id4, lower Slug, and higher E-cad levels had better survival than those showing lower Id4, higher Slug, and lower E-cad levels (Figure 4a,b, right; *p* = 0.0017 for overall survival and 0.15 for relapse-free survival).

Multivariable Cox proportional-hazards regression analyses, with a stepwise selection model, were used to evaluate the associations of various independent prognostic factors with patient overall survival (Table 1). Our results revealed that the independent prognostic factor was Id4 expression (hazard ratio (HR) = 0.61, 95% confidence interval (CI) = 0.41–0.90; *p* = 0.012). Simultaneously, the independent prognostic factors associated with relapse-free survival (Table 2) were Id4 (HR = 0.68, 95% CI = 0.54–0.86; *p* = 0.001), Slug (HR = 1.73, 95% CI = 1.25–2.38; *p* = 0.001) and E-cad (HR = 1.08, 95% CI = 1.01–1.16, *p* = 0.035) expression. Therefore, Id4 may serve as a marker for identifying high-risk patients and as a potential therapeutic target for the treatment of lung adenocarcinoma. Then, a tree diagram was created to display the conditional probabilities of Id4, Slug, and E-cadherin expressions (Figure 4c). The results indicated that 14.3% of stage I patients express high Id4/E-cadherin and low Slug expression levels. On the other hand, 11.9% of stage I patients express low Id4/E-cadherin and high Slug expression levels. Collectively, there are about 25% stage I LADC patients for which clinical outcomes could be predicted by using the series of the gene panel.

## 3. Discussion

Until now, the role of Id4 in cancer has remained ambiguous [39,40,41,42,43,44,45]. Although much evidence has suggested the different roles of Id4 in various cancers, the function of Id4 in lung cancer metastasis requires more careful analysis. Here, we found that manipulating the expression of Id4 in lung cancer cells could interfere with cell migratory and invasive abilities, regulate the occurrence of EMT through controlling the Slug/E-cadherin axis in vitro, and affect cancer metastasis in vivo. In addition, we also analyzed the gene expression data of 168 pathological stage I LADC patients from GSE31210, which is a public database that includes gene expression data of pathological stage I and II lung adenocarcinomas with basic clinical characteristics but no therapy-related data [51]. The results showed that the Id4, Slug, and E-cad signatures could be used as indicators to significantly predict the clinical outcome of 25% stage I LADC patients. Moreover, the multivariable Cox proportional-hazards regression analyses also revealed that the expression of Id4 is an independent prognostic factor for both overall (HR = 0.61, 95% CI = 0.41–0.90; *p* = 0.012) and relapse-free (HR = 0.68, 95% CI = 0.54–0.86; *p* = 0.001) survival. All these demonstrate that Id4 may act as an invasion suppressor in lung cancer progression. The data of this study, showing that Id4 may act to impede the evolution of lung cancer metastasis, are similar to those from prostate cancer [41,42], glioblastoma [44], and thyroid tumors [52]. Recently, Nasif and his colleague demonstrated that Id4 is significantly methylated in ER+ breast tumors [40]. On the other hand, Cheng et al. also found that lncRNA SNHG7 could regulate the expression of Id4 via sponging miR-342-3p in pancreatic cancer [53]. Although Id4 plays the opposite role in these cancers, whether the downregulation of Id4 in lung cancer metastasis occurs through epigenetic or non-coding RNA regulation should be further clarified in the near future.

According to our results, we could indicate that enhancing the expression of Id4 reduced the cell invasive ability in both CL1-5 and H1299 lung adenocarcinoma cell lines, for which the Id4 expression belongs to a relatively lower baseline (Figure 1c). However, the inhibition of the invasive capability in CL1-5 cell lines was weaker than in H1299 cell lines. We considered that the p53 status of cells might be an important issue. It is well-known that H1299 is a p53-null lung cancer cell line, and our research team also previously identified CL1-5 as a p53-mutant cell line [54]. We considered that the different genetic backgrounds in cell lines might manipulate fewer novel signaling pathways in each other. These specific molecular mechanisms might interfere with the role of Id4 in cancer metastasis. The results which were simulated for the invasion assay in vitro were also confirmed by a tail vein metastatic assay in vivo. We not only established an Id4-manipulating metastatic experiment in H1299 (Figure 1d) but also in CL1-5 (Figure S2). The metastasis ability in both H1299 and CL1-5/Id4-overexpressing group was down-regulated, but the metastatic degrees were different. The metastatic ability in the H1299/Id4-overexpressing group decreased about fivefold compared with the vector control, but the suppression in the CL1-5/ Id4-overexpressing group was about threefold compared with the vector control.

**Figure 5:**
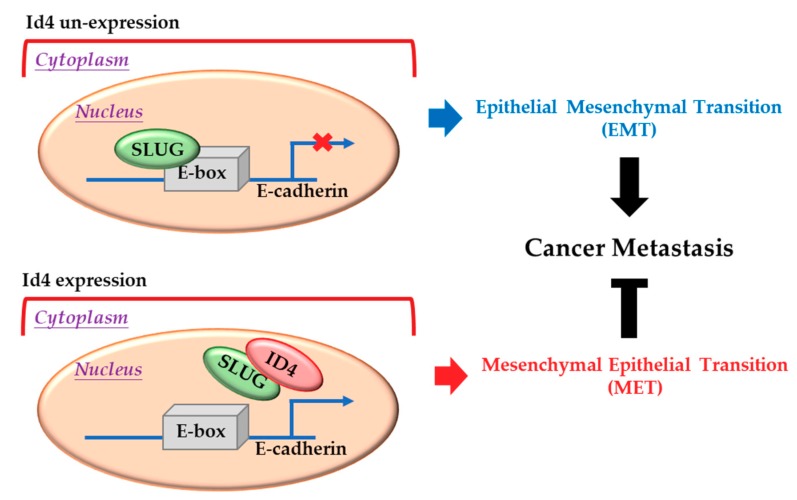
Inhibitor of DNA binding 4 (Id4) induces mesenchymal-epithelial transition (MET) through regulating the Slug/E-cadherin axis. Up: Slug protein can bind to the E-box motif in the promoter region and suppress the expression of E-cadherin in the nucleus. Bottom: When Id4 is overexpressed in cells, it can bind to Slug, remove the Slug protein from the E-box motif in the promoter region, promote the expression of E-cadherin, and induce cell-processed MET.

Usually, Id proteins function as dominant-negative transcription factors through dimerizing with a basic helix-loop-helix (bHLH) transcription factors and blocking their DNA binding activities [8,9,10,11,13]. Slug, a well-known zinc finger transcriptional repressor, has been reported to participate in epithelial–mesenchymal transition (EMT) and cancer metastasis by suppressing its downstream target genes (such as E-cadherin, occludin, claudin 1, and integrin α3) [55,56,57]. In this study, we demonstrated that Id4 could interact with Slug, inhibit its binding to the E-box (sequence: CANNTG) promoter region, induce the expression of E-cad, promote the occurrence of mesenchymal-epithelial transition, and suppress lung cancer metastasis (Figure 5). Moreover, we also found that the expression of Id4 could significantly down-regulate the transcriptional expression of Slug in lung cancer cells. These findings are similar to the report of Rahme and colleagues, which revealed that Id4 could down-regulate the expression of MMP-2 through inhibitory interaction with Twist in glioblastoma [44]. As Snail and Slug are downstream targets of Twist1, the phenomena of Id4-induced Slug down-regulation at a transcription level may be regulated by Twist1.

In our results, we found that the existence of Id4 could dramatically affect the expression of E-cad, but the suppressive promoter activities of Slug could only be recovered by nearly 20% when cells co-expressed Id4. As such, we could not rule out the possibility that in addition to Slug, there may exist other candidates that also participate in Id4-mediated EMT/MET regulation. For example, E47, one of the E2A proteins, has previously been reported to promiscuously associate with all members of the Id protein family, including Id4 [58]. Previously, Shin Kim et al. also reported that the hepatitis B virus X protein (HBx) could induce epithelial–mesenchymal transition (EMT) by repressing E-cadherin expression via the upregulation of E12/E47 [59]. This evidence suggests the possibility that E47 may also be involved in the regulation of the Id4-mediated MET process. Whether E47 or any other bHLH transcription factors participate in inhibiting lung cancer metastasis through Id4 should be further explored.

## 4. Materials and Methods

### 4.1. Cell Lines and Culture Condition

The human lung adenocarcinoma cell lines, CL1-0 and CL1-5, with differing cell invasiveness, were kind gifts from Professor Pan-Chyr Yang (National Taiwan University, Taipei, Taiwan), who established the cell lines via a Transwell invasion chamber in 1997 [47]. The human lung cancer cells A549 (ATCC^®^ CCL-185™), H1975 (ATCC^®^ CRL-5908™), H3255 (ATCC^®^ CRL-2882™), and H1299 (ATCC^®^ CRL-5803™), and the human bronchial epithelial cell BEAS2B (ATCC^®^ CRL-9609™), were purchased from the American Type Culture Collection (ATCC, Gaithersburg, MD, USA). All the cell lines were cultured in Roswell Park Memorial Institute (RPMI) 1640 or Dulbecco’s modified Eagle’s medium (DMEM) (Invitrogen; Thermo Fisher Scientific, Inc., Waltham, MA, USA) containing 10% fetal bovine serum (FBS) (Invitrogen), 1% penicillin, streptomycin, and 1 mM sodium pyruvate (all from Sigma-Aldrich, St. Louis, MO, USA) at 37 °C in a humidified atmosphere of 5% CO_2_. Cells were detached from the culture plates using 0.1% trypsin-0.05% ethylenediaminetetraacetic acid (EDTA; Sigma-Aldrich).

### 4.2. Reverse Transcription Polymerase Chain Reaction (RT-PCR) and Microarray Analysis

Cells were homogenized and RNA was isolated using TRIzol reagent (Invitrogen), according to the manufacturer’s instructions. Total RNA was reverse transcribed to cDNA and the gene expressions were examined by a polymerase chain reaction, as previously described [60]. The sequences of oligonucleotide primers are listed in Table S2. Moreover, the original OneArray (Phalanx Biotech, Hsinchu, Taiwan) analyses of CL1-5/Id4 overexpression and vector control stable cells were performed by the Phalanx Biotech Group and functional annotations using the Ingenuity Pathway Analysis Knowledge Base (http://www.ingenuity.com/).

### 4.3. Plasmid Constructs and Stable Cell Selection

The cDNA encoding full-length human Id4 (GeneBankTM accession number NM_001546.3), and Slug (NM_003068.4) and its deletion mutants, were PCR-amplified. Then, the expression plasmids produced were transferred by ligation PCR-generated inserts into pcDNA3.1/V5-His-TOPO (Invitrogen) and pFlag-CMV-2 (Sigma-Aldrich) vectors. To establish CL1-5/Id4-overexpressing or vector control stable cells, purified pcDNA3.1-V5-Id4 or pcDNA3.1-V5-His-TOPO plasmids were transfected into 70% confluent CL1-5 cells using Lipofectamine2000 transfection reagents (Invitrogen) in a total volume of 1 mL Opti-MEM (Invitrogen), as previously described [7]. Then, Geneticin (G418; Merck, Darmstadt, Germany) was added at a concentration of 600 ug/mL to select for a pooled population of stable transfectants, and the selection medium was changed every 3 d for another 3 weeks. Clones of resistant cells were isolated and allowed to expand for further characterization.

### 4.4. Short Hairpin RNA (shRNA) and Lentiviral Infection

The plasmids, pLKO.1-shLacz and pLKO.1-shId4, were obtained from the National RNAi Core Facility Platform (Academia Sinica, Taipei, Taiwan). Following previously described procedures [7], HEK293T cells were transfected with pLKO.1-shLacZ or pLKO.1-shId4, together with two helper plasmids, pCMVDR8.91 and pMD.G, using Lipofectamine 2000 transfection reagent (Invitrogen). The viruses were harvested at 24, 48, and 72 h after transfection; filtered using a 0.45 μm low-protein-binding filter; and frozen at −80 °C. The cells were transduced in the presence of polybrene (8 mg/mL, Sigma) with lentiviral particles at a multiplicity of infection (MOI) of 2. Twenty-four hours after infection, the cells were treated with puromycin (final concentration 0.75 μg/mL) and puromycin-resistant clones were selected and pooled.

### 4.5. Immunoprecipitation and Immunoblotting

Immunoprecipitation was performed as described previously [7]. In brief, transfected cells were lysed on ice for 5–10 min in immunoprecipitation (IP) lysis buffer (20 mM Tris pH 8.0, 150 mM NaCl, 100 μM Na_3_VO_4_, 50 mM NaF, 30 mM sodium pyrophosphate, and 0.5% NP-40; all from Sigma), and a 25-fold dilution of stock solution was treated with one Mini Protease Inhibitor Cocktail Tablet (Roche Diagnostics, Mannheim, Germany) dissolved in 2 mL of distilled water. The cell lysates were passed several times through a 21-gauge needle and clarified by centrifugation at 12,000 rpm for 30 min at 4 °C. The supernatants were taken as the total cell lysates. V5-tagged Id4 or Flag-tagged Slug was immunoprecipitated using anti-V5 or anti-Flag antibodies and protein A-Sepharose beads (Invitrogen). The precipitated proteins were separated by SDS-PAGE and transferred to polyvinylidene membranes (Millipore, Billerica, MA, USA) for immunoblotting with anti-Flag (1:5000), anti-V5 (1: 10000), anti-Id4 (1:1000), anti-Slug (1: 5000), anti-E-cad (1:5000), anti-N-cad (1:5000), anti-β-actin (1:20000), and anti-α-tubulin (1:20000) primary antibodies, followed by appropriate secondary antibodies conjugated with horseradish peroxidase, and signals were detected by Chemiluminescent Substrates (PerkinElmer, Waltham, MA, USA).

### 4.6. Cell Proliferation and Apoptosis

A trypan blue exclusion assay was used to determine cell proliferation. Briefly, cells were seeded in 24 well plates (10^4^ cells/well) for 24, 48, and 72 h, and then cell number was then counted with trypan blue solution (Sigma). A FITC Annexin V Apoptosis Detection Kit (BD-Pharmingen^TM^) was used to examine cell apoptosis. Cells were seeded in 10 cm culture plates (1.8 × 10^6^ cells) for 48 hours and treated with Annexin V solution, and the result was detected by flow cytometry.

### 4.7. Modified Boyden Chamber Invasion Assay

Modified Boyden chambers with polycarbonate-membrane inserts (pore size, 8 μm; Falcon; Becton Dickinson, Franklin Lakes, NJ) coated with 30 μg of Matrigel (BD; San Jose, CA, USA) were used for cell invasion assays. Stable transfectants were suspended in RPMI containing 10% NuSerum (Invitrogen). Cells (2.5 × 10^4^) were placed in the upper chambers, and 1 mL of medium was placed in the lower chambers. After incubation for 18 h at 37 °C, the cells were fixed with methanol for 10 min at room temperature and then stained for 30 min at room temperature with 10% Gemisa Stain solution (Sigma). The number of cells on each membrane was counted under a microscope at 100× magnification. All experiments were performed at least twice, and each sample was assayed in triplicate.

### 4.8. Luciferase Reporter Assay

The Slug binding region containing three tandem repeats of the Snail-binding site (SBS; 5’-AGC TTA GCA GGT GCA CGA TAT CAG CAG GTG CAC CAT ATG AGC AGG TGC AA-3’) and E-cadherin promoter sequences were described as has been done previously [48,61]. Cells cultured in 6 well plates were transfected using Lipofectamine 2000, according to the manufacturer’s protocol. Thirty-six hours after transfection, cell extracts were prepared using the reporter lysis buffer, and luciferase activity was assessed using the dual luciferase reporter assay system (Promega, Madison, WI, USA) and a luminometer, according to the manufacturer’s instructions. A control reporter expressing Renilla luciferase was used for normalization of the transfection efficiency.

### 4.9. Chromatin Immunoprecipitation Assay (ChIP)

The assays were performed using a Magna ChIP™ A/G Chromatin Immunoprecipitation Kit (Millipore), according to the manufacturer’s instructions. Briefly, equal numbers of cells were treated with 1% formaldehyde and then quenched with 0.125 M glycine for protein-DNA cross-linking. After being washed with cold phosphate buffered saline (PBS), the cells were scraped, and soluble chromatin lysates were extracted via sonication and centrifugation. Two percent of the diluted chromatin solution was reserved as the total input sample. The diluted chromatin solution was incubated with anti-Slug and normal goat immunoglobulin G (goat IgG) antibodies overnight at 4 °C with rotation. Then, the DNA/protein solution was eluted with proteinase K containing elution buffer at 65 °C for 2 h to break the formaldehyde cross-links. The DNA solution was used as the template for 33 cycles of PCR amplification using E-cadherin gene-specific primers (forward: 5’-CGAACCCTGTGGAATCAGAA-3’; reverse: 5’-GCGGGCTGGAGTCTGAACTG-3’).

### 4.10. Experimental Metastasis In Vivo

The in vivo tail vein metastasis assay was performed as described previously [7]. Briefly, a single-cell suspension containing 10^6^ cells (H1299 or CL1-5/vector and H1299 or CL1-5/Id4-overexpressing cells) in 0.1 mL PBS was injected into the lateral tail veins of 6-week-old SCID mice (*n* = 10 per group, supplied by the National Laboratory Animal Center, Taipei, Taiwan). Ten weeks after injection, the relevant mice were sacrificed by carbon dioxide anesthesia, and their lungs were removed, weighed, and fixed in 10% formalin. Additionally, the number of metastatic nodules was counted under a dissecting microscope. Embedded tissues were sliced into 4 μm sections, and the sections were stained with hematoxylin-eosin for histological analysis. All mouse experiments were performed in accordance with the animal guidelines and with the approval of the Laboratory Animal Center, National Taiwan University College of Medicine (IACUC number: 20140034, 26 February 2014 for date of approval).

### 4.11. IHC Analysis of Tumor Samples from the Lungs of Mice

The sections used for IHC analysis of ID4, SLUG, E-cadherin, and Ki67 protein expression were first autoclaved in Antigen Retrieval Citra Solution (BioGenex, Milmont Dr, Fermont, CA, USA) at 121 °C for 10 min. The samples were then treated with 3% H_2_O_2_; and sequentially, subjected to incubation with 0.1% BSA for 1 hour, and then with a monoclonal anti-ID4 antibody (Santa Cruz Biotechnology, Dallas, TX, USA; 1:100), a polyclonal anti-SLUG antibody (Santa Cruz Biotechnology; 1:100), a monoclonal anti- E-cadherin (BD; 1:100), and a rabbit polyclonal anti-Ki67 (Abcam, Cambridge, UK; 1:500) overnight at 4 °C. Detection of the immunostaining was performed using the Super Sensitive Non-Biotin Polymer HRP Detection System (BioGenex), according to the manufacturer’s instructions.

### 4.12. TUNEL Assay

The sections used for in situ apoptosis detection analysis were first treated with 3% H_2_O_2-_methanol. Detection of the apoptosis was performed using the in situ apoptosis detection kit (Trevigen, Gaithersburg, MD, USA), according to the manufacturer’s instructions.

### 4.13. Statistical Analysis

Data were presented as the means and their standard errors (SEs). The statistical analyses were performed by Student’s t-test and the survival curves were obtained by the Kaplan–Meier log-rank test with the SPSS Statistical Program (v10.0; SPSS, Inc., Chicago, IL, USA). All statistical tests were two- sided, and a *p* value of <0.05 was considered statistically significant. A multivariable Cox proportional-hazards regression model was fitted with the following variables: continuous age, sex, epidermal growth factor receptor (EGFR) status, Lysyl-TRNA Synthetase (KARS) status, Id4 expression (high versus low), Slug expression (high versus low), and E-cadherin expression (high versus low).

## 5. Conclusions

Overall, our study showed that Id4 is a key factor in controlling Slug-mediated epithelial–mesenchymal transition. The expression of Id4 could inhibit the binding of Slug in the E-box promoter region, significantly increase the expression of E-cad, and suppress cancer metastasis in human lung cancer. Moreover, we also showed that the signatures of Id4 and Slug could be used as good indicators to predict the clinical outcome of LADC patients. These findings may provide a new area for developing novel lung cancer therapeutics in the near future.

## Figures and Tables

**Table 1:** Hazard ratios (HRs) for death (from any cause) among patients with LADC, according to multivariable Cox regression analysis ^A^.

Variable	HR (95% CI)	*p*
Age	1.00 (0.94–1.07)	0.93
Gender (male as reference)	0.91 (0.26–3.22)	0.88
Smoking	1.02 (0.27–3.80)	0.98
EGFR	0.45 (0.15–1.31)	0.14
KRAS	028 (0.03–2.29)	0.23
Id4 expression (per 1000 +)	0.61 (0.41–0.90)	0.012
Slug expression (per 1000 +)	1.32 (0.86–2.02)	0.20
E-cad expression (per 10 +)	0.97 (0.85–1.12)	0.69

^A^ Stepwise selection was used to choose the optimal multivariable Cox proportional-hazards regression model. Id4, Slug, and E-cad expression were designated as continuous variables. *p*-values (two-sided) were calculated using a *χ*^2^ test. CI: confidence interval.

**Table 2:** Hazard ratios (HRs) for death (from relapse) among patients with LADC, according to multivariable Cox regression analysis ^A^.

Variable	HR (95% CI)	*p*
Age	1.01 (0.96–1.05)	0.83
Gender (male as reference)	1.06 (0.43–2.60)	0.89
Smoking	0.82 (0.32–2.07)	0.67
EGFR	0.49 (0.23–1.06)	0.069
KRAS	0.53 (0.17–1.68)	0.28
Id4 expression (per 1000 +)	0.68 (0.54–0.86)	0.001
Slug expression (per 1000 +)	1.73 (1.25–2.38)	0.001
E-cad expression (per 10 +)	1.08 (1.01–1.16)	0.035

^A^ Stepwise selection was used to choose the optimal multivariable Cox proportional-hazards regression model. Id4, Slug, and E-cad expression were designated as continuous variables. *p*-values (two-sided) were calculated using a *χ*^2^ test.

## Supplementary Materials

The following are available online at https://www.mdpi.com/2072-6694/11/12/2021/s1. Figure S1: The correlation between Id4 expression and invasive ability in CL cell lines, Figure S2: The effects of Id4 expression in cancer metastasis in vivo were examined by a tail vein metastasis assay with CL1-5/Id4-overexpressing stable cells, Figure S3: The examinations of cell proliferation and the apoptotic population in vitro and in vivo, Figure S4: The expression of EMT markers in metastatic pulmonary nodules, Figure S5: The results of RT-PCR (**a**) and blots (**b**) from Figure S1b, Figure S6: The results of RT-PCR (**a**) and blots (**b** and **c**) from Figure 1a and Figure 1b, Figure S7: The results of RT-PCR from Figure 2a and Figure 2., Figure S8: The blots from Figure 2a and Figure 2d, Figure S9: The blots from Figure 3a (**a**) and Figure 3b (**b**), Figure S10: The blots from Figure 3c (a) and Figure 3d (b), Figure S11: The blots (a) and the results of ChIP from Figure 3e, Table S1: The clinical characteristics of 168 stage I lung adenocarcinomas LADC patients in the public database GSE31210, Table S2: The sequence of the oligonucleotide primers for RT-PCR.
