# Supplementary material for: Inhibitor of DNA-Binding Protein 4 Suppresses Cancer Metastasis through the Regulation of Epithelial Mesenchymal Transition in Lung Adenocarcinoma
Source: Cancers (Basel). 2019 Dec 14;11(12):2021. doi: 10.3390/cancers11122021 (PMC6966672; doi:10.3390/cancers11122021)
Supplement: Supplementary file 1 [file cancers-11-02021-s001.pdf]

Supplementary Materials: Inhibitor of DNA-Binding Protein 4 Suppresses Cancer Metastasis through the Regulation of Epithelial Mesenchymal Transition in Lung Adenocarcinoma

Chi-Chung Wang, Yuan-Ling Hsu, Chi-Jen Chang, Chia-Jen Wang, Tzu-Hung Hsiao and Szu-Hua Pan

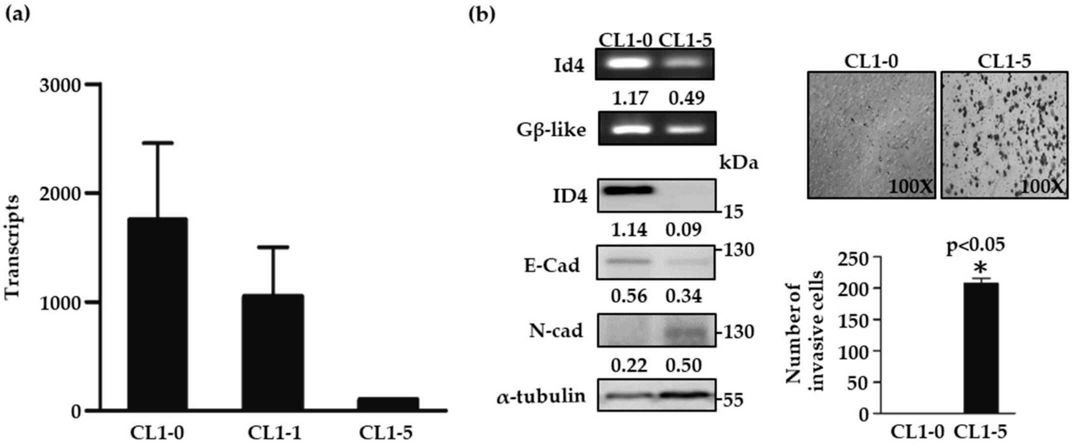

Figure S1. The correlation between Id4 expression and invasive ability in CL cell lines. (**a**) The mRNA expressions of Id4 in CL1-0, CL1-1, and CL1-5 cell lines by cDNA microarray analysis. (**b**) Id4 mRNA and protein expression (left) levels in CL1-0, and CL1-5 lines were detected by RT-PCR and immunoblotting. The invasive ability of each cell line was evaluated by a modified Boyden chamber invasion assay in vitro. The images of the invasion assay (original magnification, ×100) were presented (right) and the number of invasive cells was calculated (bottom left, * $p < 0.05$). The protein expressions of E-cadherin and N-cadherin were also presented by immunoblotting. The quantification of mRNA and protein expressions were calculated by ImageJ, and normalized with the internal control, Gβ-like or α-tubulin, of each cell line.

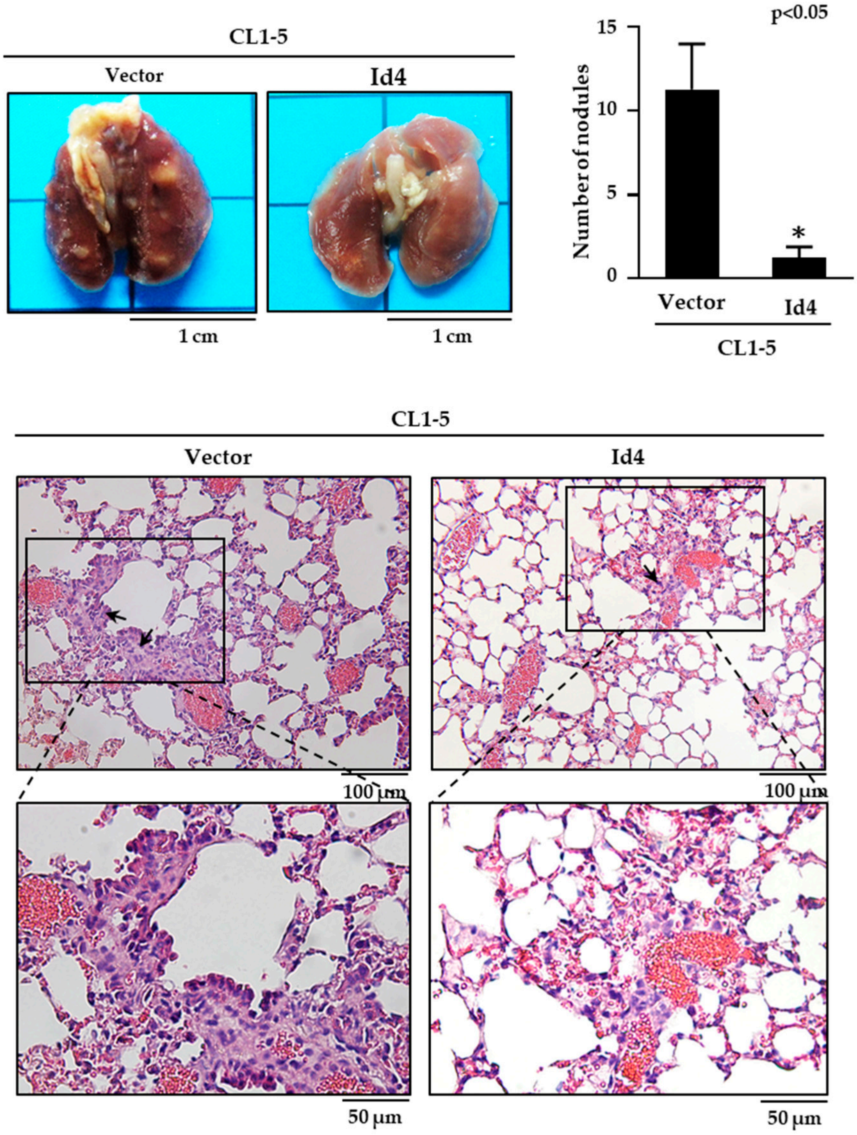

Figure S2. The effects of Id4 expression in cancer metastasis in vivo were examined by a tail vein metastasis assay with CL1-5/Id4-overexpressing stable cells. Numbers of metastatic tumor nodules were calculated from four mice per group (* $p < 0.05$). Histology of the metastatic pulmonary nodules were confirmed as LADC by H&E staining; the arrows indicated the distribution of tumors and the area of black rectangles were zoomed and presented in the bottom.

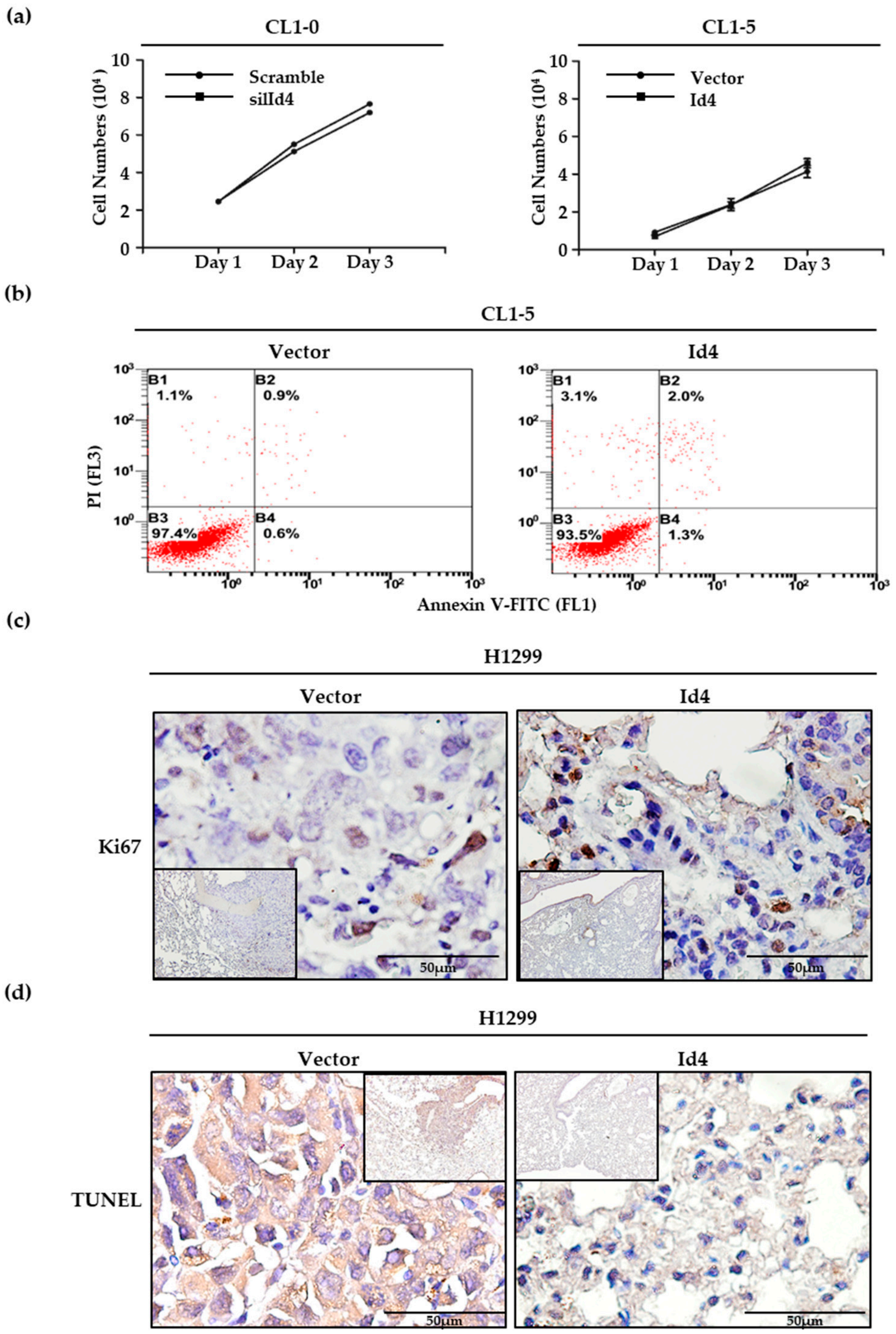

Figure S3. The examinations of cell proliferation and the apoptotic population in vitro and in vivo. (**a**) The proliferation ability of CL1-0/Id4-silneing (left) and CL1-5/Id4-overexpressing (right) cells were calculated by trypan blue exclusion assay. Numbers of cells were calculated from 6 replicates well per group. (**b**) The cell apoptosis was measured by FITC Annexin V Apoptosis Detection Kit in CL1-5/Id4-overexpressing stable cell lines and analyzed as percentage. (c and d) The detection of proliferation and apoptosis in vivo. Immunohistochemistry staining of Ki67 (**c**), and TUNEL staining (**d**) were obtained from mice, which were tail vein injected with H1299/Id4-overexpressing stable cells. The images were taken, and the location of Ki67 or the apoptosis in cells were presented in the nucleus with brown color.

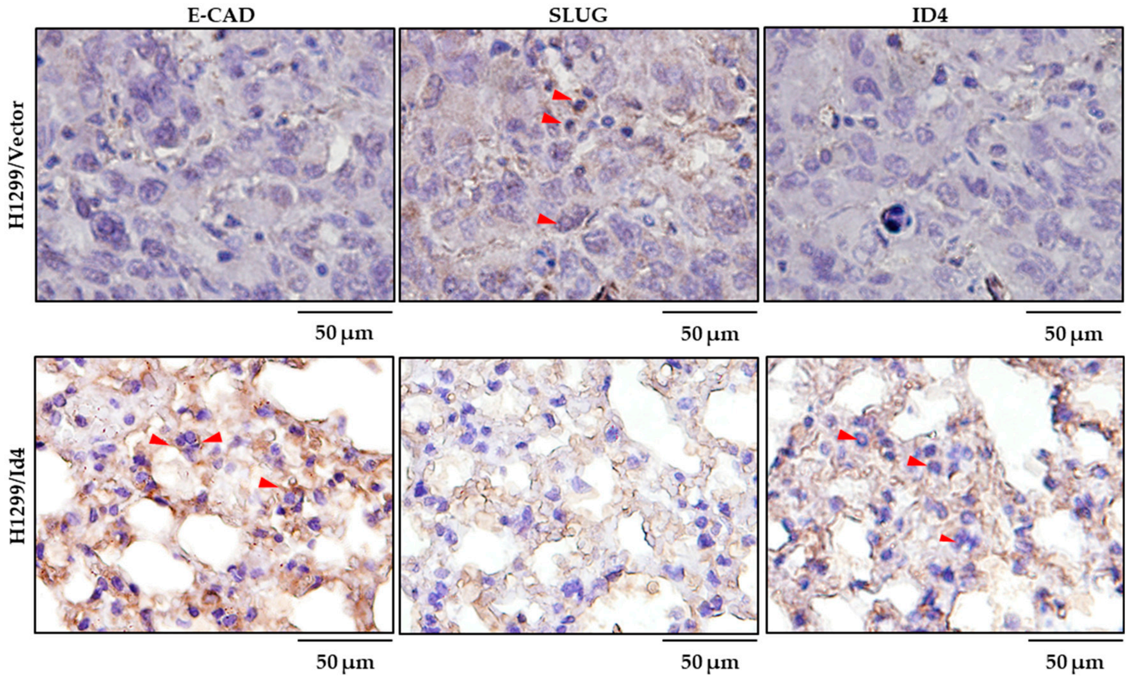

Figure S4. The expression of EMT markers in metastatic pulmonary nodules. Immunohistochemistry staining (IHC staining) of E-cadherin, SLUG, and ID4 in tumor sections of lung tissue was obtained from mice, which were tail vein injected with H1299/Id4-overexpressing stable cells. The images were taken, and the location of protein is indicated in the figure (red arrow heads).

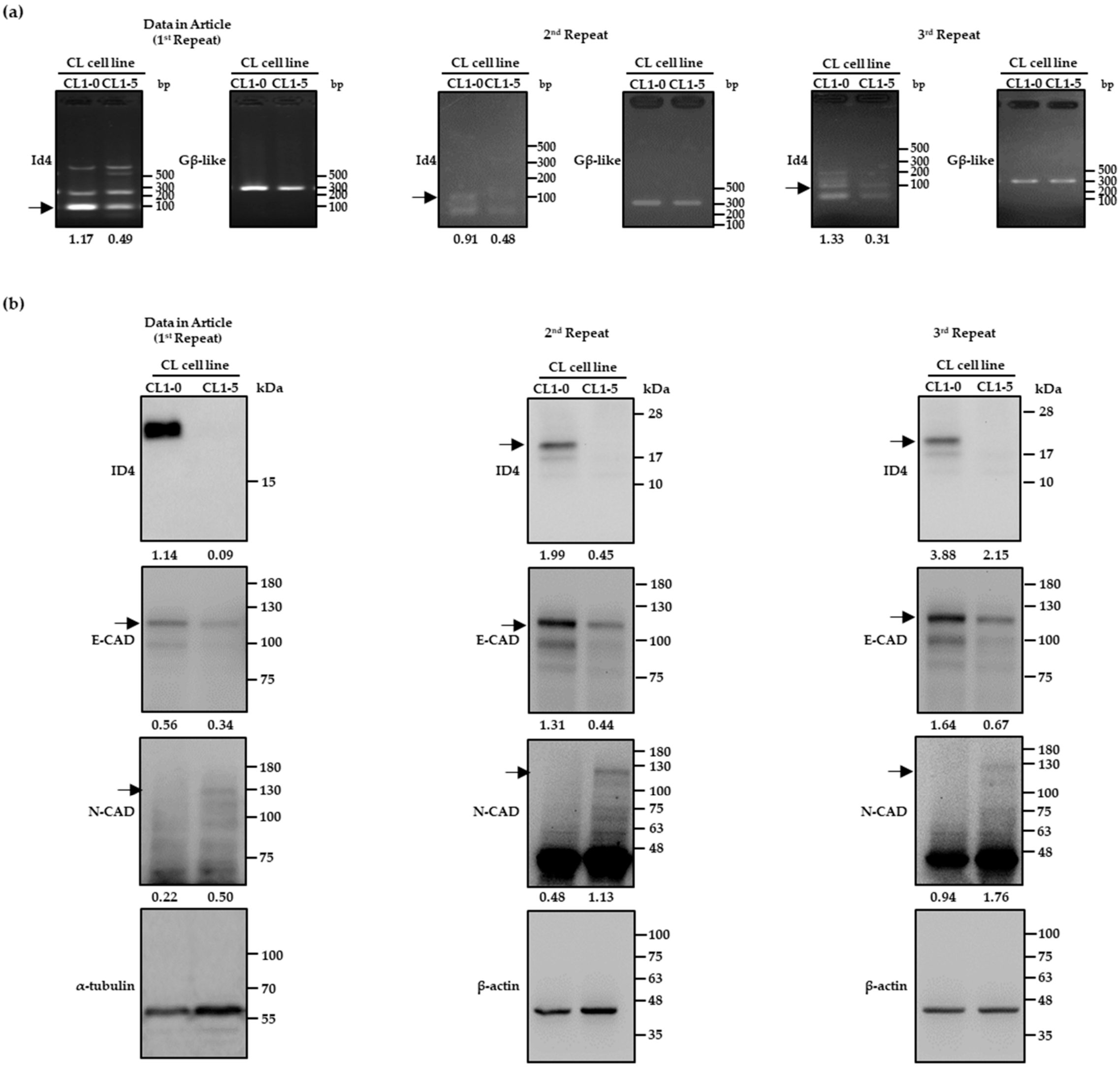

Figure S5. The results of RT-PCR (**a**) and blots (**b**) from supplementary Figure 1b. The numbers under the images of bands indicated the quantification of mRNA and protein expressions, which were calculated by ImageJ, and normalized with the internal control, Gβ-like, α-tubulin or β-actin, of each cell line.

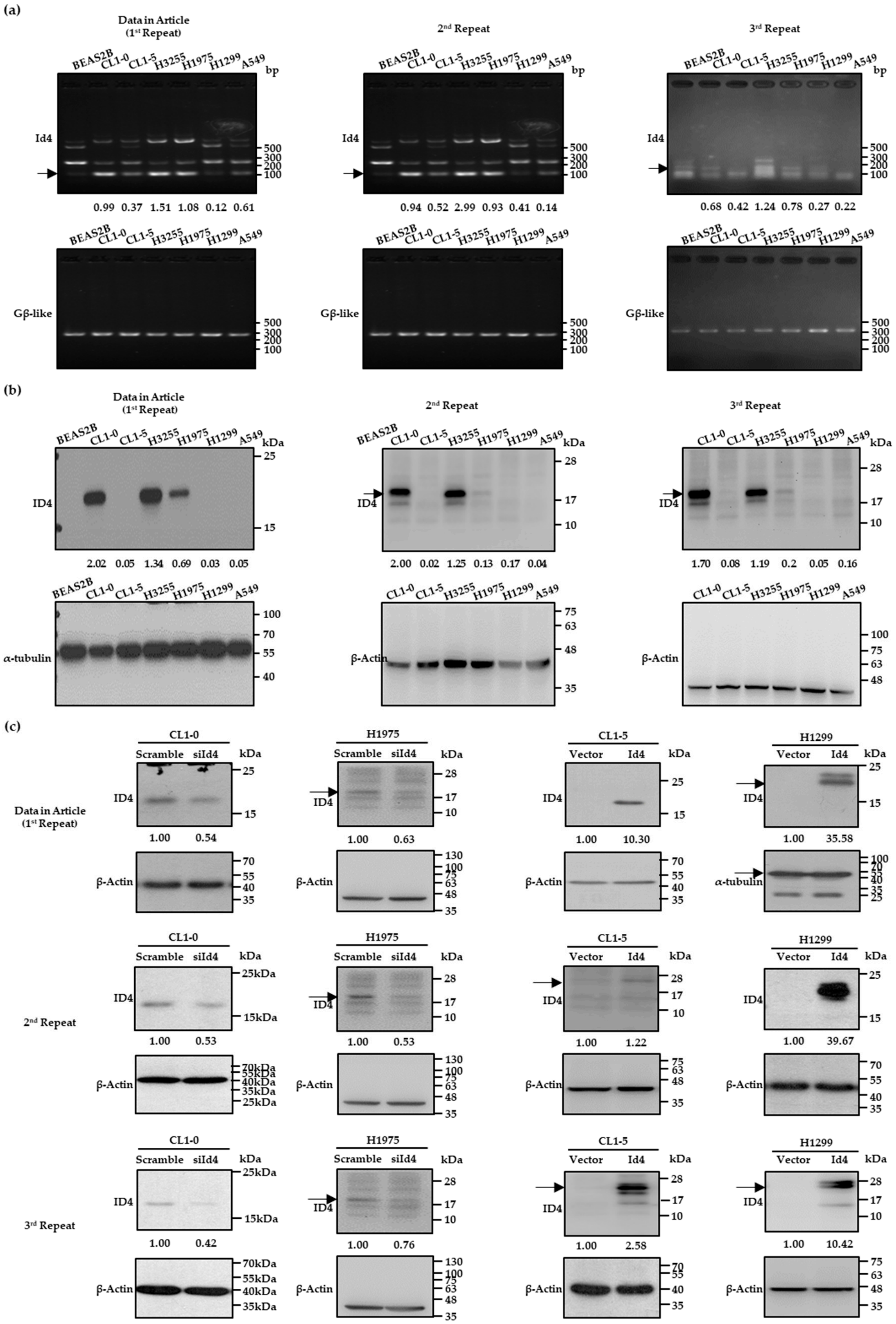

Figure S6. The results of RT-PCR (**a**) and blots (**b** and **c**) from Figure 1a and Figure 1b. The numbers under the images of bands indicated the quantification of mRNA and protein expressions, which were calculated by ImageJ, and normalized with the internal control, Gβ-like,

α-tubulin or β-actin, of each cell line (**a** and **b**). (**c**) The protein expression levels in Id4-overexpressing or silencing cells were quantified and displayed the relative fold changes compared with the scramble or vector control cells.

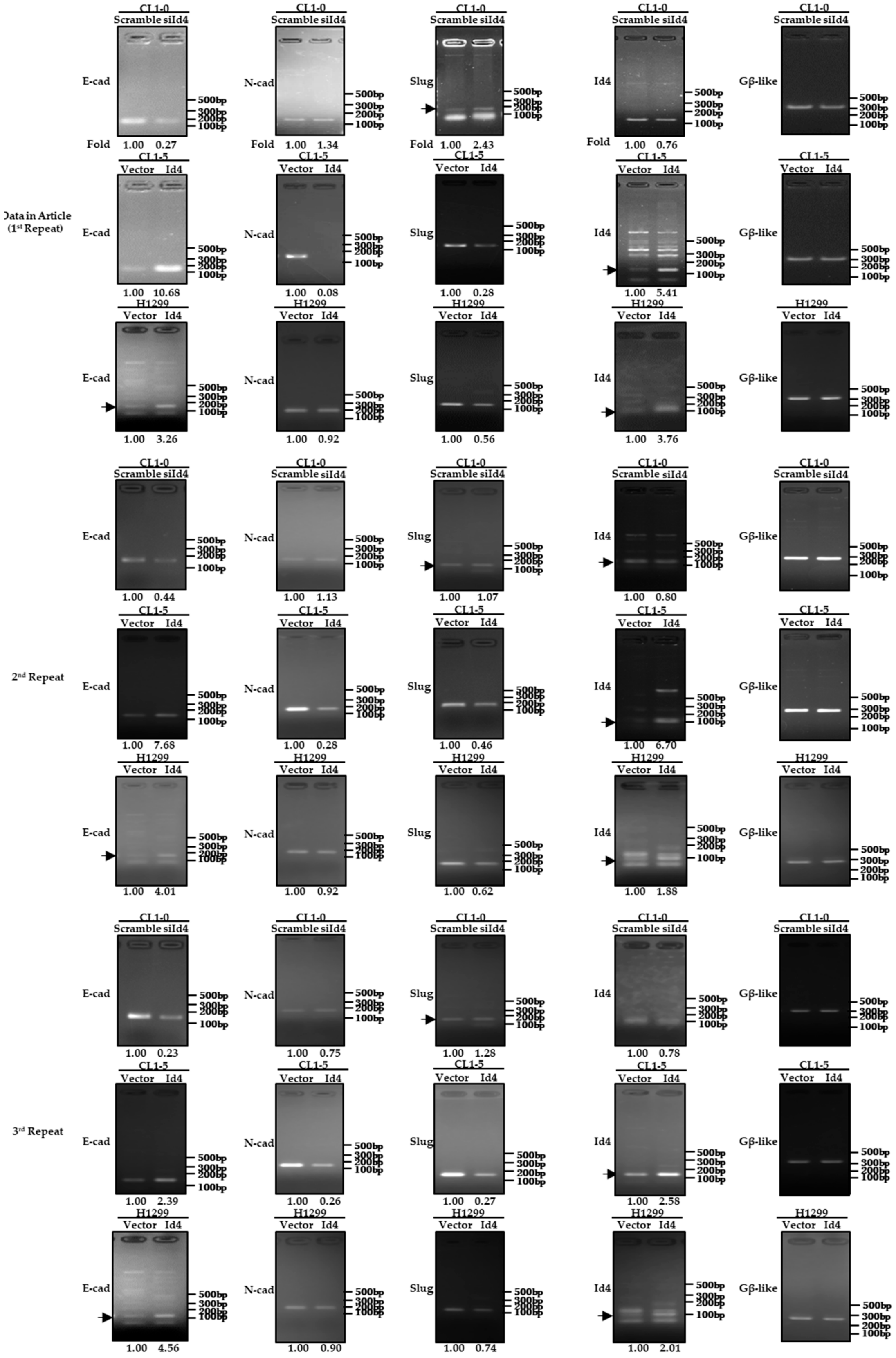

Figure S7. The results of RT-PCR from Figure 2a and Figure 2d. The mRNA expression levels in Id4-overexpressing or silencing cells were quantified and displayed the relative fold changes compared with the scramble or vector control cells. The images of Id4 and Gβ-like in Figure 2d were occurred in the secondary repeat.

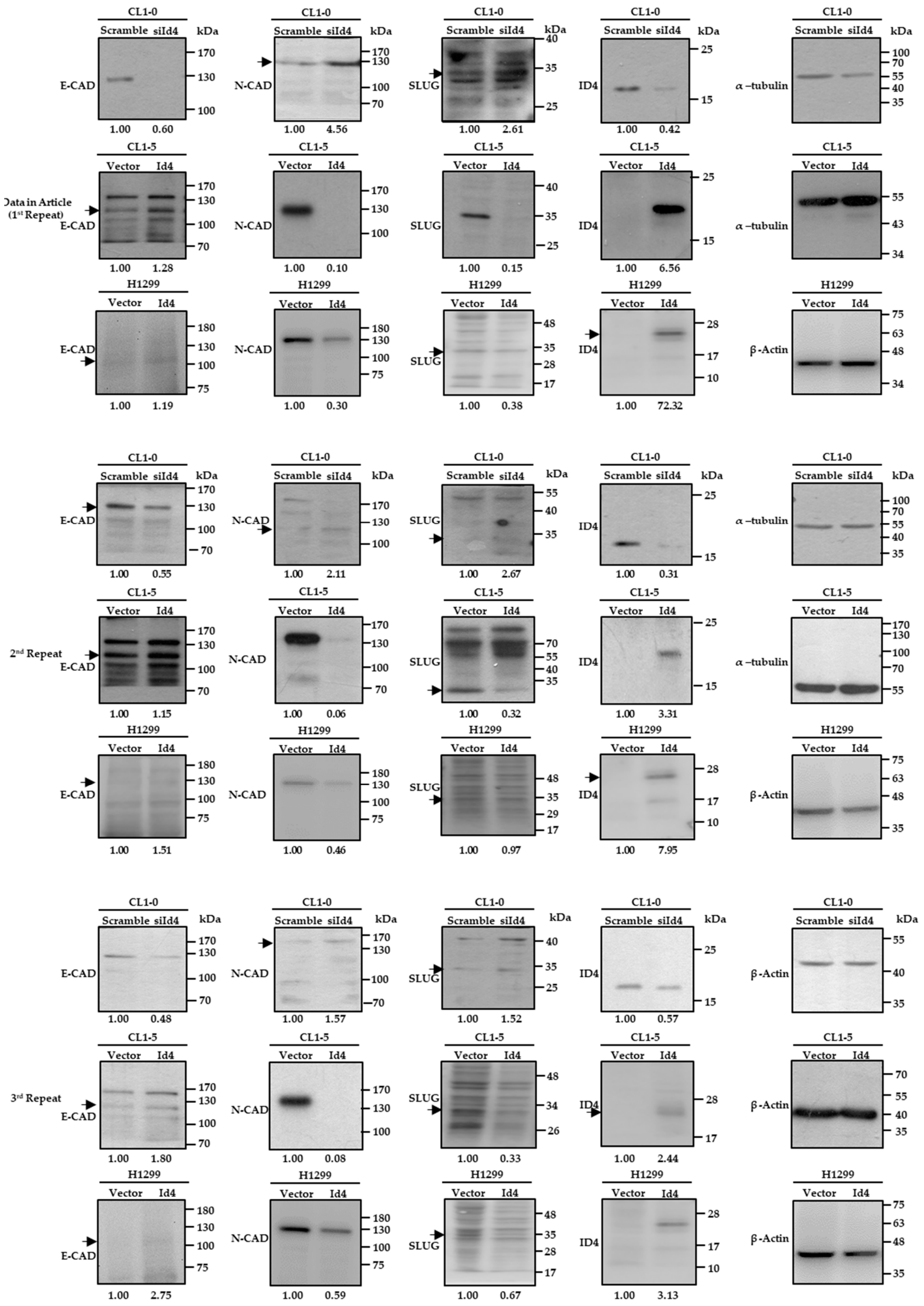

Figure S8. The blots from Figure 2a and Figure 2d. The protein expression levels in Id4-overexpressing or silencing cells were quantified and displayed the relative fold changes compared with the scramble or vector control cells. The images of ID4 and internal control (α-tubulin or β-actin) in Figure 2d were occurred in the secondary repeat.

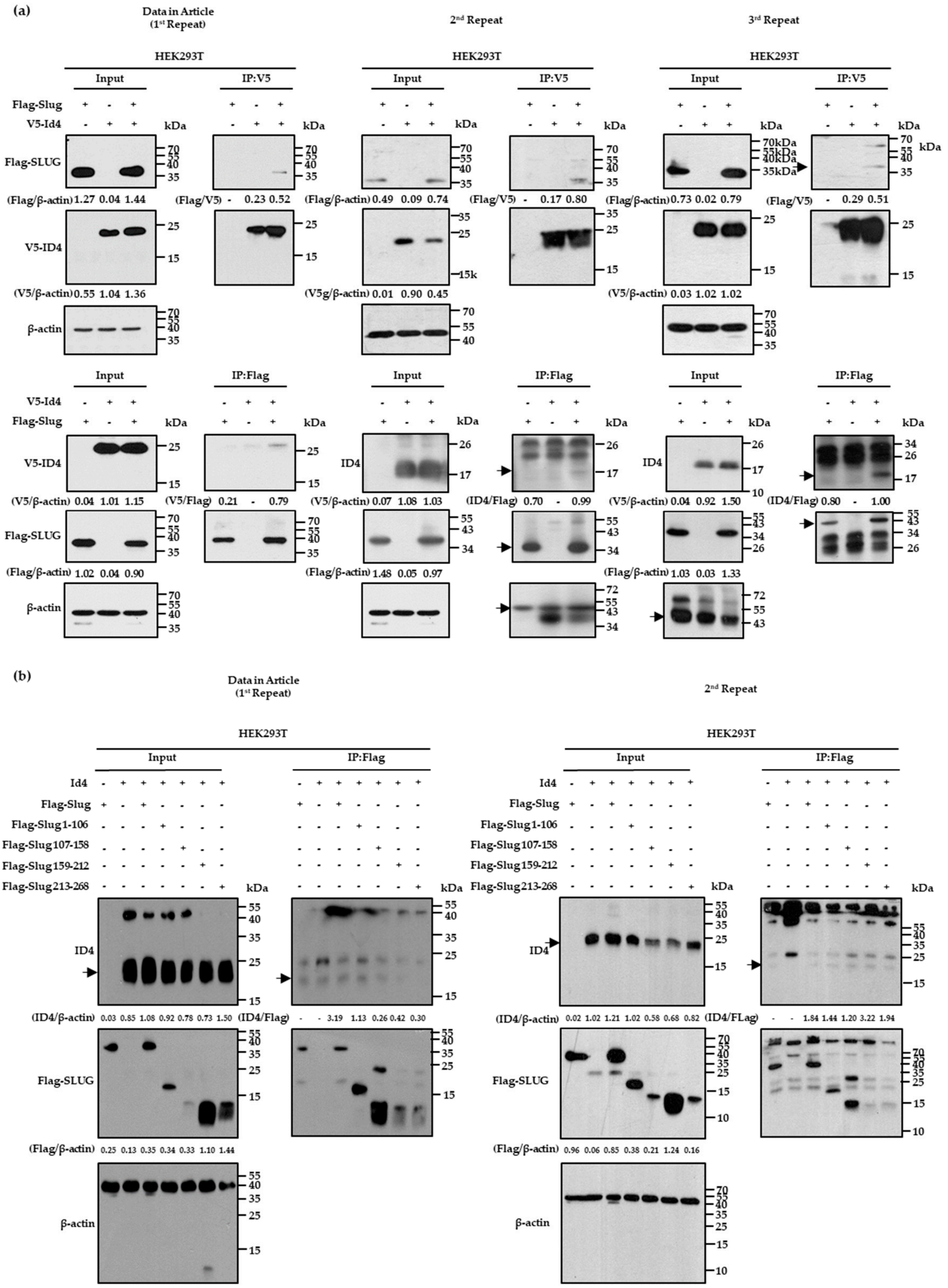

Figure S9. The blots from Figure 3a (**a**) and Figure 3b (**b**).

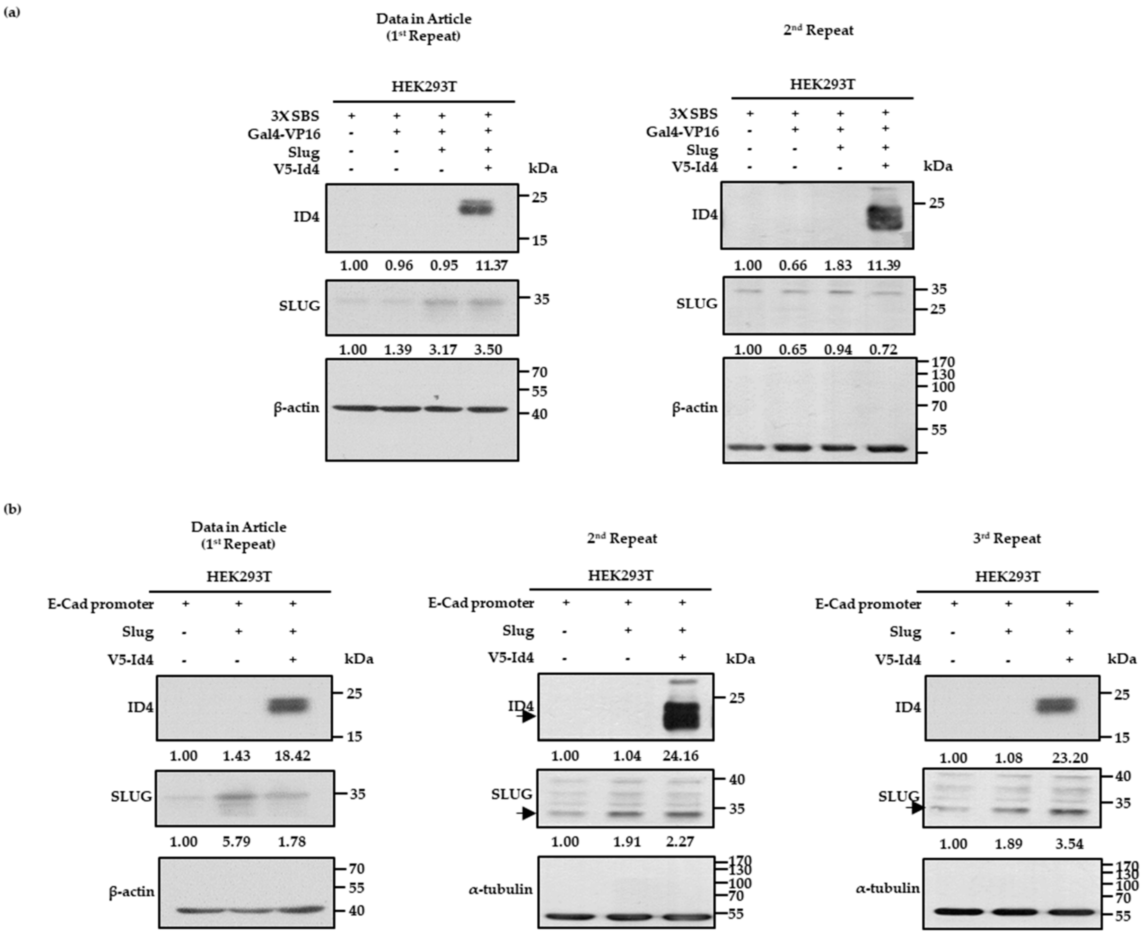

Figure S10. The blots from Figure 3c (**a**) and Figure 3d (**b**). The protein expression levels in cells were quantified and displayed the relative fold changes compared with the control cells, which were only transfected with promoter-construction.

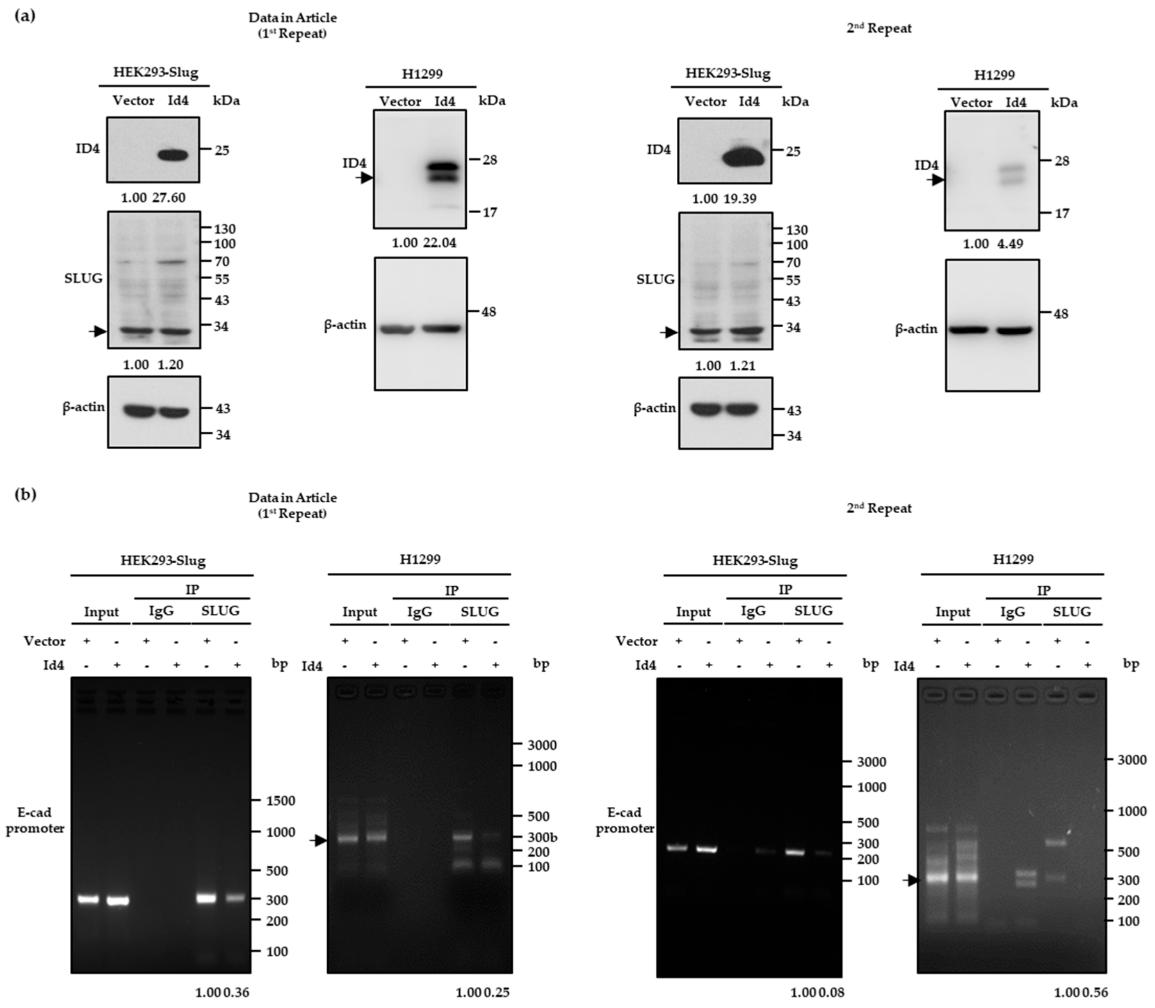

Figure S11. The blots (**a**) and the results of ChIP from Figure 3e. (**a**) The protein expression levels in Id4-overexpressing cells were quantified and displayed the relative fold changes compared with the vector control cells. (**b**) The DNA expression levels in Id4-overexpressing cells were quantified with the vector control cells.

Table S1. The clinical characteristics of 168 stage I lung adenocarcinomas LADC patients in the public database GSE31210 [51].

Characteristics	High Id4 Expressions Patient No. (%)	Low Id4 Expressiosn Patient No. (%)	*p*	High Slug Expressions Patient No. (%)	Low Slug Expressiosn Patient No. (%)	*p*	High E-cad Expressions Patient No. (%)	Low E-cad Expressiosn Patient No. (%)	*p*
**Age** (mean ± SD)	60.7 ± 6.7	59.1 ± 7.4	0.14 [a]	61.1 ± 6.8	58.4 ± 7.1	0.01 [a]	59.6 ± 7.3	60.5 ± 6.9	0.30 [a]
**Sex**									
Female	34 (40.5)	37 (44.0)	0.75 [b]	38 (45.2)	33 (39.3)	0.53 [b]	52 (61.9)	39 (46.4)	0.06 [b]
Male	50 (59.5)	47 (56.0)		46 (54.8)	51 (60.7)		32 (38.1)	45 (53.6)	
**Smoking**									
Yes	30 (35.7)	44 (52.4)	0.04 [b]	38 (45.2)	36 (42.9)	0.88 [b]	39 (46.4)	35 (41.7)	0.64 [b]
No	54 (64.3)	40 (47.6)		46 (54.8)	48 (57.1)		45 (53.6)	49 (58.3)	
**Relapse**									
Yes	9 (10.7)	28 (33.3)	<0.01 [b]	23 (27.4)	14 (16.7)	0.14 [b]	22 (26.2)	15 (17.9)	0.26 [b]
No	75 (89.3)	56 (66.7)		61 (72.6)	70 (83.3)		62 (73.8)	69 (82.1)	

**Gene Alteration Status**									
ALK-fusion +	2 (2.4)	1 (1.2)	0.57[c]	2 (2.4)	1 (1.2)	0.02 [c]	2 (2.4)	1 (1.2)	0.30 [c]
EGFR mutation +	54 (64.3)	49 (58.3)		43 (51.2)	60 (71.4)		57 (67.9)	46 (54.8)	
KRAS mutation +	5 (6.0)	9 (10.7)		9 (10.7)	5 (6.0)		17 (20.2)	6 (7.1)	
EGFR/KRAS/ALK -	23 (27.4)	25 (29.8)		30 (35.7)	18 (21.4)		8 (9.5)	31 (36.9)	

[a] Student's t-test; [b] Fisher's exact test; [c] Chi-square test.

Table S2. The sequence of the oligonucleotide primers for RT- PCR**.**

Name		Sequence (5′→3′)	Product Size (bp)
Id4	F	AACAAGCAGGGCGACAGC	129
	R	CTCCGGTGGCTTTTTTTCTCT	
E-cadherin	F	CCCAATACATCTCCCTTCACAG	121
	R	CCACCTCTAAGGCCATCTTTG	
N-cadherin	F	CTCCTATGAGTGGAACAGGAACG	150
	R	TTGGATCAATGTCATAATCAAGTGCTGTA	
Slug	F	GCTGCCAAATCATTTCAACTG	148
	R	CCAGACAACCGACATGTAATG	
G -like	F	GTATGGAACCTGGCTAACTG	306
	R	TACTGATAACTTCTTGCTTC	

© 2019 by the authors. Licensee MDPI, Basel, Switzerland. This article is an open access article distributed under the terms and conditions of the Creative Commons Attribution (CC BY) license (http://creativecommons.org/licenses/by/4.0/).
